# Sterilization of Silver Nanoparticles Using Standard Gamma Irradiation Procedure Affects Particle Integrity and Biocompatibility

**DOI:** 10.4172/2157-7439.S5-001

**Published:** 2011-10-25

**Authors:** Jiwen Zheng, Jeffrey D. Clogston, Anil K. Patri, Marina A. Dobrovolskaia, Scott E. McNeil

**Affiliations:** Nanotechnology Characterization Laboratory, Advanced Technology Program, SAIC-Frederick, Inc., NCI-Frederick, Frederick, MD21702

**Keywords:** Silver nanoparticles, Gamma irradiation, Autoclave, Sterilization, Particle size, Hematocompatibility

## Abstract

Silver nanoparticles are commonly used in a variety of commercial and medical products. Here we investigate the effects of standard sterilization methods, including heat/steam (autoclave) and gamma-irradiation on the structural integrity and biocompatibility of citrate-stabilized silver nanoparticles with nominal sizes of 20, 40, 60 and 80 nm. Particle size, shape and in vitro biocompatibility were studied pre- and post-sterilization. Sterilization by gamma irradiation at dose levels commonly used in medical device industry (15, 25 and 50 kGy) resulted in dramatic changes in particle size and morphology, as monitored by dynamic light scattering (DLS) and transmission electron microscopy (TEM). Exposing the particles to a chemical producer of hydroxyl radicals (N-hydroxy-2-pyridinethione) allowed us to duplicate the sterilization-based changes in size and morphology, implying a free radical mechanism of action. Compared to untreated controls, we also observed a three- to five-fold increase in tendency of sterilized silver nanoparticles to cause platelet aggregation, a sensitive in vitro indicator of thrombogenicity.

## Introduction

Silver nanoparticles are appearing with ever-increasing frequency in consumer products, with over 300 self-identified nanosilver-containing products on markets [1]. These include dispersions and powders marketed as antimicrobials (e.g. for the treatment of foods, swimming pools, washing machine liners) and products infused with nanosilver (e.g. clothes, wound dressings, “self-sanitizing” toothbrushes, packaging and cosmetics). Other examples of medical products containing nanosilver include the Acticoat Wound Care with Nanocrystalline Silver (FDA approved in 1996), which prevents wound infections, and the I-Flow Silver Soaker Nanosilver Catheter (FDA approved in 2005).

As novel nanosilver is incorporated into an increasing number of products subject to FDA regulation, questions about formulation, pyrogenicity, sterility, and sterilization procedures are emerging. For example, very little is known about the stability of silver nanoparticles under standard sterilization procedures commonly used in the food industry, and for medical devices and drug formulations. These effects require thorough investigation, since any beneficial effects derived from the nanoscale size of silver particles could be reduced or eliminated if their integrity is compromised when the product is sterilized.

While many studies have focused on the benefits of using silver nanoparticles [2-6], none, to our knowledge, address the sterilization stability of silver nanoparticles. Standard sterilization methods include heat/steam (autoclave), ethylene oxide (EtOx) and various forms of gamma irradiation [7-9]. The sterilization stability of nanoparticle based formulations may depend on many factors including their composition, surface reactivity, and thermal stability. Sterilization by gamma irradiation, for instance, is known to be destructive to certain pharmaceuticals and water- and oil-based formulations [10]. Published studies evaluating heat/steam sterilization and gamma irradiation effects on polymeric nanoparticle stability concluded that polymer-based nanoparticles do not tolerate heat/steam sterilization or high doses of gamma irradiation [11-13]. Low doses of gamma irradiation and high doses delivered from a low energy source, however, were well tolerated by most studied nanoparticles [12,14-17]. Citrate-stabilized colloidal gold nanoparticles developed by the National Institutes of Standards and Technologies (NIST) as nanoparticle reference materials (RM 8011, 8012 and 8013) were sterilized by 32 kGy gamma irradiation [18] with no observed change in their physicochemical properties (including size).

In this paper, we investigate the effects of two types of sterilization methods: autoclave (heat/steam treatment) and three doses of gammairradiation traditionally used in medical device industry (15, 25 and 50 kGy) [7,8] on the structural integrity and biocompatibility of silver nanoparticles. Our test materials included commercially-available citrate-stabilized silver nanoparticles with nominal particle sizes (diameters) of 20, 40, 60 and 80 nm. These sizes were selected because they are representative of those used in commercial products and share the most common preparation/synthesis procedure used for nanosilver.

## Materials and Methods

### Materials

Citrate-stabilized silver nanoparticles (20, 40, 60 and 80 nm nominal size) were purchased from Ted Pella, Inc. (Redding, CA). We did observe considerable variability in the advertised size and the measured size (by DLS and TEM, Table 1). Organic solvents chloroform and ethanol were purchased from Aldrich. N-hydroxy- 2-pyridinethione (HPT) was purchased from Aldrich. UV Lamp (Pen Ray^®^ UV lamp, 254 nm, 4400 μW/cm^2^) and power supply were purchased from UVP, Inc. (San Gabriel, CA) and used as the energy source to produce hydroxyradicals in a solution of HPT. Quartz mirco cuvette (120 μL, 10 mm pathlength) was bought from Hellma. The amine functionalized silicon Smart grids for TEM measurement were purchased from Dune Sciences, Inc. (Eugene, OR) (for detailed information please refer to http://www.saramostafavi.com/dune/dune/whygrid.html). Regular TEM carbon/formvar copper grids were purchased from EMS (Hatfield, PA). NIST standard silver reference material SRM 3151 was purchased from the National Institute of Science and Technology (NIST) (Gaithersburg, MD).

### Dynamic Light Scattering (DLS)

A Malvern Zetasizer Nano ZS instrument (Southborough, MA) with back scattering detector (173°C, 633 nm laser wavelength) was used for measuring the hydrodynamic size (diameter) in batch mode at 25°C in a low volume quartz cuvette (pathlength 10 mm). Samples were prepared by diluting stock solutions 10-fold in water. Hydrodynamic size is reported as the intensity-weighted average over all size populations (Z-avg), and as the intensity-weighted and volume-weighted average over a particular range of size populations corresponding to the most prominent peak in the % intensity and % volume distributions (Int-Peak and Vol-Peak), respectively. Traces in the figures represent the average of at least twelve measurements. Filtration of the autoclaved sample through a 0.1 μm syringe filter did not affect the size as monitored by DLS, suggesting there were no large aggregates which would be removed by the filter (data not shown).

### Sterilization procedures

Gamma irradiation from a Cobalt-60 source was conducted at the United States Army Medical Research Institute for Infectious Diseases (USAMRIID), according to ISO standard 11137-2, Sterilization of Health Care Products. The exposure rate was 6829 roentgen/min and the exposure times were as follows: 3.75 hours to deliver 15 kGy, 6.2 hours to deliver 25 kGy, and 12.5 hours to deliver 50 kGy. The temperature was monitored during exposure and did not exceed 30°C. Pre and post gamma irradiation, the particles were stored at a nominal temperature of 4°C (2-8°C), and sterilization was conducted at room temperature (20-23°C). Heat/steam sterilization was conducted according to ANSI/AAMI standard 79-2006 and included particles exposure to 121°C for 30 minutes in a gravity displacement autoclave.

### Transmission Electron Microscopy (TEM)

TEM images were obtained using a Hitachi H7600 instrument operated at 80kV. A 400 mesh formvar carbon-coated copper grid was glow discharged in a vacuum evaporator (Denton) for 30 seconds in order to make the grid hydrophilic, and thus attractive to particles. A2 μL aliquot of silver colloid particle solution was dropped onto the grid and incubated for 30 seconds. After the 30 second incubation, excess sample was wicked off with filter paper and allowed to air-dry prior to TEM imaging.

### Immobilization of silver nanoparticles on silicon-based smart TEM grids

Amine-terminated silicon-based Smart TEM grids were first cleaned by rinsing with chloroform, ethanol and milli-Q water, then blown-dry with a N_2_ stream. A 3 μL aliquot of colloidal silver was dropped onto the grid and incubated for 1 minute, after which, the silver drop was blotted off with a piece of filter paper and allowed to air-dry before TEM imaging.

### HPT treatment of silver nanoparticle

N-hydroxy-2-pyridinethione (HPT) was added to 18 MΩcm Milli-Q H2O to make a 0.6 mM stock solution, For silver colloid treatment, an aliquot of 2, 5, or 10 μL of HPT stock solution was added to 600 μL of silver colloid solution. The sample vials were then exposed to UV light at a distance of 5 cm for 1, 2, or 3 h, and the UV-vis spectra of each sample was recorded; reported values are normalized to the untreated control.

### UV-vis spectroscopy measurement

The UV-vis measurement was conducted using a Perkin Elmer Lambda 35 spectrometer. An aliquot of stock citrate-stabilized nanoparticles were added to a 100 μL microquartz cuvette, and the spectra were recorded between 300 nm and 700 nm. The scan speed was 960 nm/min and the data interval was 1 nm. Milli-Q water was used as both the reference and blank for baseline subtraction. The surface plasmon resonance (SPR) absorption peak of the Ag nanoparticle (NP) was compared before and after sterilization treatment.

### Human blood compatibility test

For the platelet aggregation study, healthy volunteer blood specimens were drawn under NCI-Frederick Protocol OH99-C-N046. Blood was collected in BD vacutainer tubes containing sodium citrate as an anticoagulant; specimens from at least three donors were pooled. Whole blood was centrifuged for 8 minutes at 200×g to obtain Platelet Rich Plasma (PRP). This PRP was treated with silver nanoparticle test material, PBS (negative control), or collagen (positive control) for 15 minutes at 37°C. After incubation, single platelet count was conducted using a Z2 counter and size analyzer (Beckman Coulter). A decrease in single platelet count, due to the platelet aggregation, was used to calculate percent aggregation. A more detailed protocol is available at http://ncl.cancer.gov/NCL_Method_ITA-2.pdf. Each sample was analyzed in duplicate and repeated 3 times. Mean result is shown (N=6).

## Results and Discussion

### Gamma irradiation, but not autoclaving, results in changes in particle integrity and hematocompatibility

**Figure 1:**
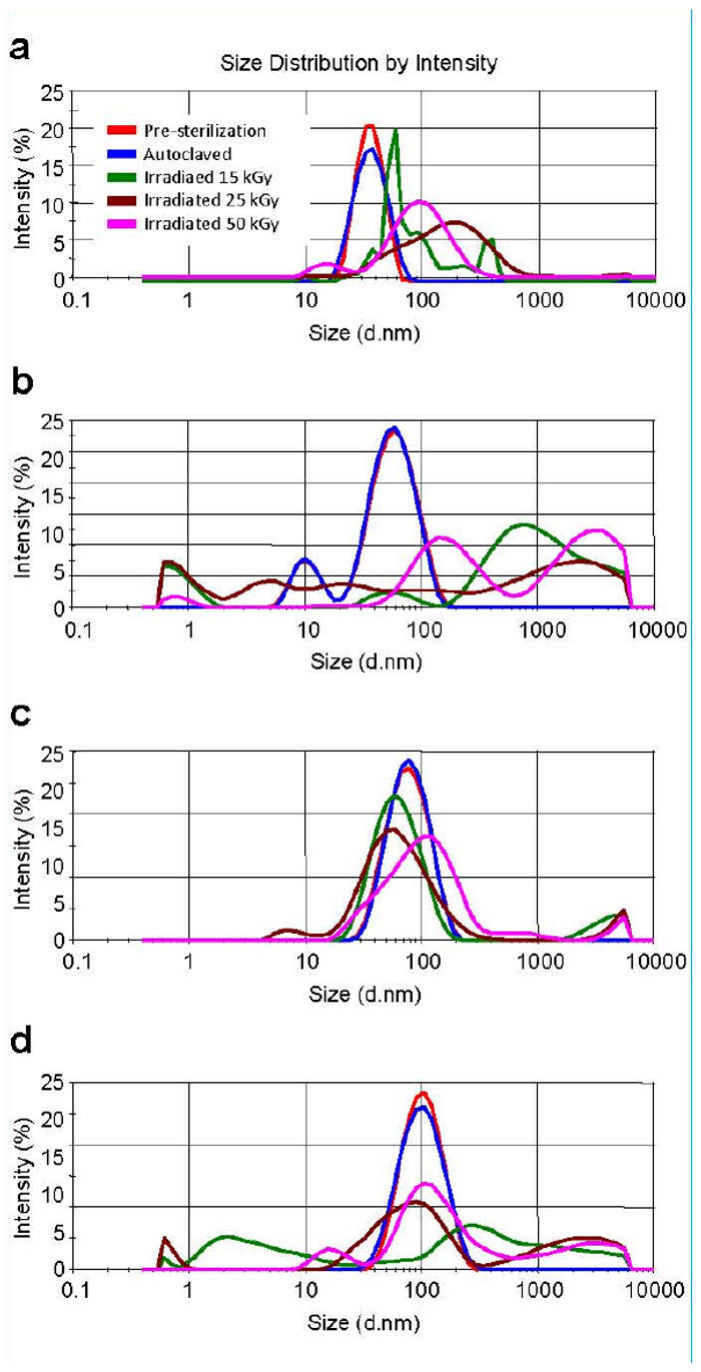
Hydrodynamic size of silver nanoparticles with nominal size 20 nm (A), 40 nm (B), 60 nm(C), and 80 nm (D) before and after gamma-irradiation

**Figure 2:**
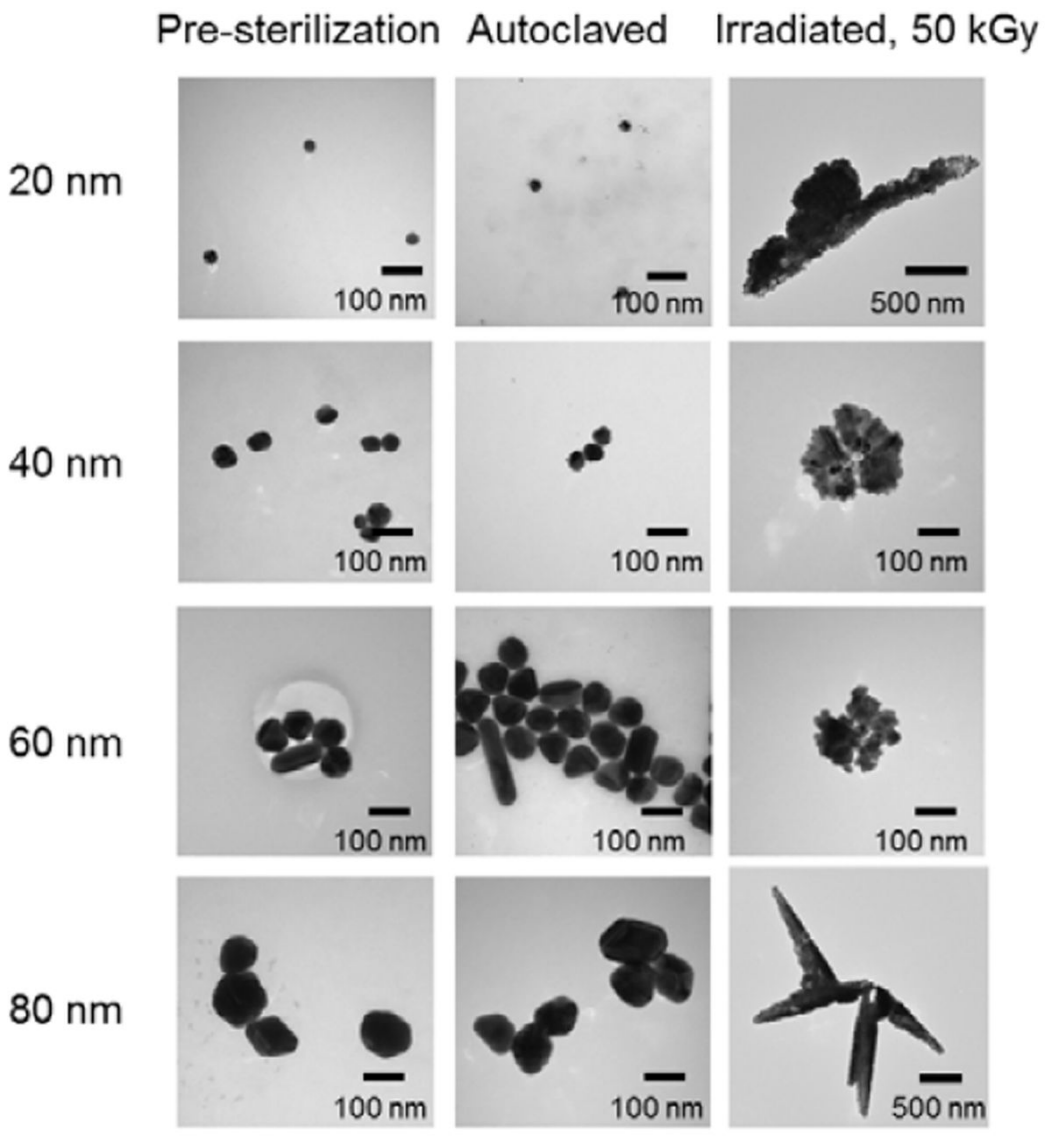
Representative TEM images of silver nanoparticles (20, 40, 60, and 80 nm) before and after sterilization treatments. The sterilization included autoclave and gamma irradiation at 3 different doses (15, 25 and 50kGy). Shown are results from irradiation at 50kGy, other doses resulted in similar changes. All scale bars are 100 nm unless otherwise noted.

Silver nanoparticles, both before and after sterilization treatments, were analyzed by dynamic light scattering (DLS) and transmission electron microscopy (TEM) to monitor size and morphology. Table 1 lists the nominal particle sizes and DLS-measured and TEM-measured particle diameters, Figure 1 shows DLS-measured size distributions, and Figure 2 contains representative EM micrographs taken before and after autoclave and irradiation sterilization.

**Figure 3:**
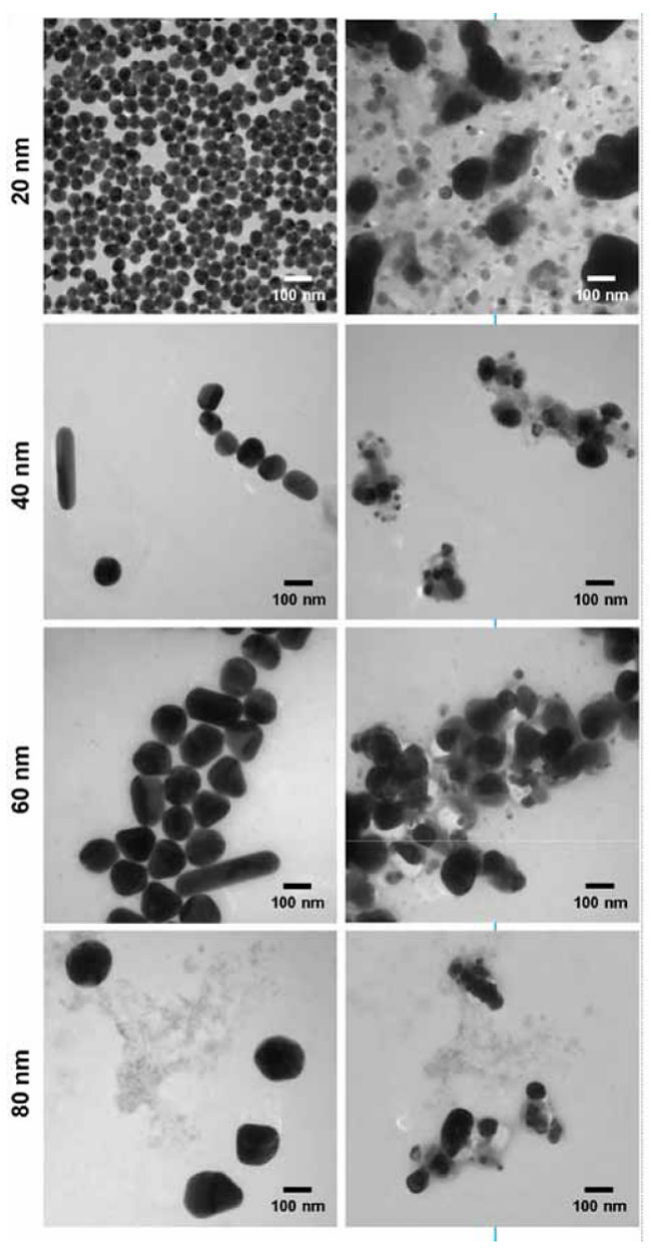
Representative TEM images of 20 nm, 40 nm, 60 nm, and 80 nm silver nanoparticles on silicon Smart TEM grids, before and after gamma irradiation at 50 kGy. TEM data clearly show gamma irradiation induced degradation of the silver colloids, resulting in both small particles and large aggregates.

There was no significant DLS-measured size difference between the untreated and post-autoclave samples for any of the 20, 40, 60, or 80 nm silver nanoparticles (Figure 1). This is in marked contrast to the irradiated particles. After gamma irradiation at any of the three dose levels, the samples were too polydispersed to make reliable size measurements by DLS. TEM measurements also showed no significant difference in size or morphology between the untreated and autoclaved samples for any of the four studied silver nanoparticles (Figure 2). TEM analysis of the irradiation-treated particles, however, revealed the morphology of the particles had been substantially altered by the sterilization. Before irradiation, all four studied nanoparticles showed typical polycrystalline features. After gamma irradiation, most particles appeared to have lost their faceted crystalline structure and formed a combination of much smaller particulates and large irregular-shaped aggregates. The large aggregates included some “sheet” structures from 100 nm to over 1 micron in length. Since sterilization of the particles was done in water as these particles were supplied by manufacturer, we questioned the role of water and storage vials. To address this question sterilization was performed in borosilicate glass tubes and in polypropylene tubes, however no differences were noted (data not shown). To allow TEM analysis of the same field of view before and after sterilization we also prepared samples where silver colloids were placed on TEM smart grids and imaged before and after gamma irradiation. Figure 3 highlights the effect of 50 kGy irradiation on the particles on TEM smart grids showing the same field of view for each nanoparticle size both before and after sterilization. We did not observe a conclusive dose-response relationship with respect to the strength of gamma irradiation. That is, the irradiated samples displayed no morphology changes that appeared to be proportional to the irradiation dose. It is plausible that all three tested irradiation energies were too high to discriminate a dose-response, as the exposures may have been greater than some putative activation energy for the morphology change.

**Figure 4:**
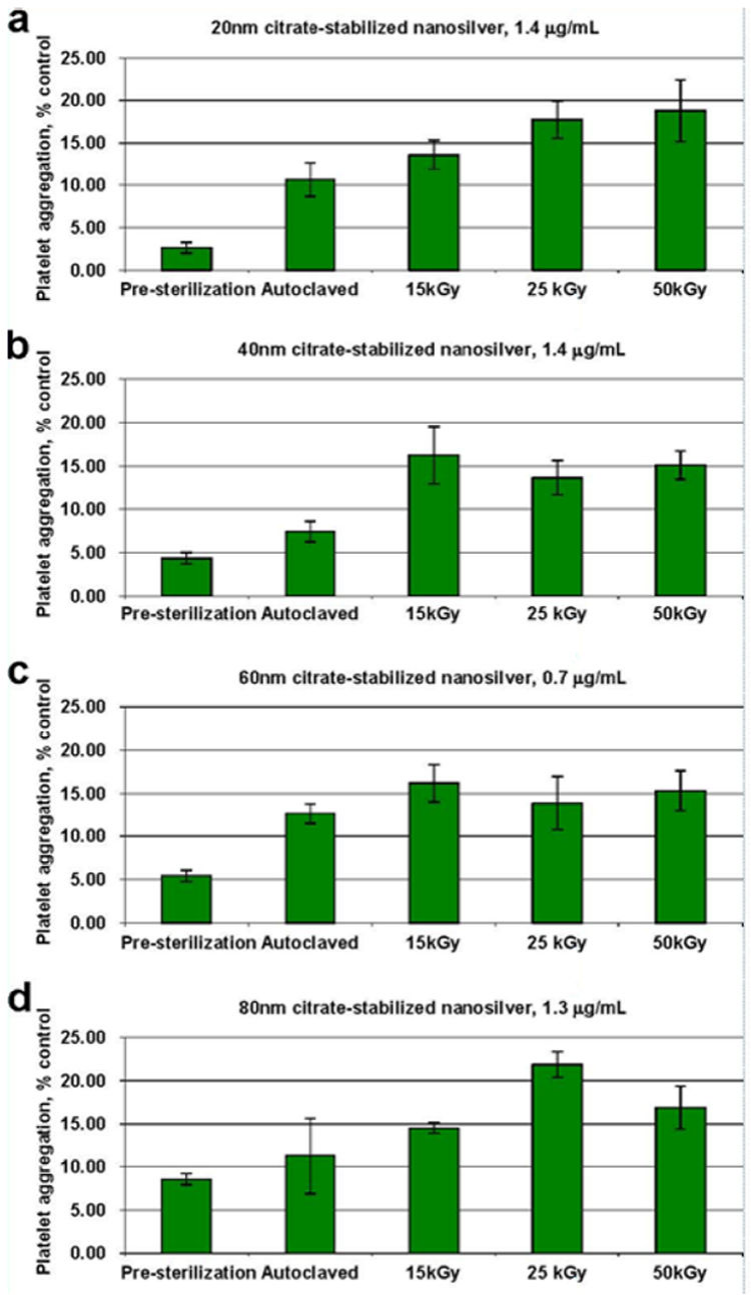
Analysis of silver nanoparticles in platelet aggregation test. Silver nanoparticles with nominal size of 20 nm (A), 40 nm (B), 60 nm (C) and 80 nm (D), untreated or sterilized with various methods, were evaluated for their potential effects on the cellular component of the blood coagulation cascade. For each nanoparticle formulation, three independent samples were prepared and analyzed in duplicate (%CV < 20). Each bar represents the mean response of these three independent samples plus SD.

In addition to investigating effects of sterilization on particle size and morphology, we also investigated the *in vitro* biocompatibility of the various silver nanoparticles before and after sterilization treatments. We and others have previously reported that platelet aggregation is a sensitive *in vitro* indicator of thrombogenicity [19]. Sterilization of all four nanoparticle samples by autoclave and all doses of gamma irradiation resulted in an up to five-fold increase in the tendency to cause platelet aggregation, at parts-per-thousand concentrations (Figure 4). No significant *in vitro* effects were observed for pre- or post-sterilized silver nanoparticles, with respect to hemolysis, complement activation, or cytotoxicity to porcine kidney cells (data not shown).

### Generation of free radicals during irradiation is responsible for particle morphology change

It is known that metal nanoparticles, such as silver and gold, can be prepared from their corresponding metal salts in a field of gamma irradiation [20]. The mechanism of this radiation synthesis of nanoparticles is not fully understood, but it is generally accepted that gamma irradiation induces ionization and excitation of the aqueous solvent, generating radiolytic molecular and radical species -- such as solvated electrons (e^-^), hydroxyl radicals (OH^•^), and hydrogen atoms (H^•^). The solvated electrons and hydrogen atoms reduce the metal ions to metal atoms, which eventually coalesce to form nanoparticles.

**Figure 5:**
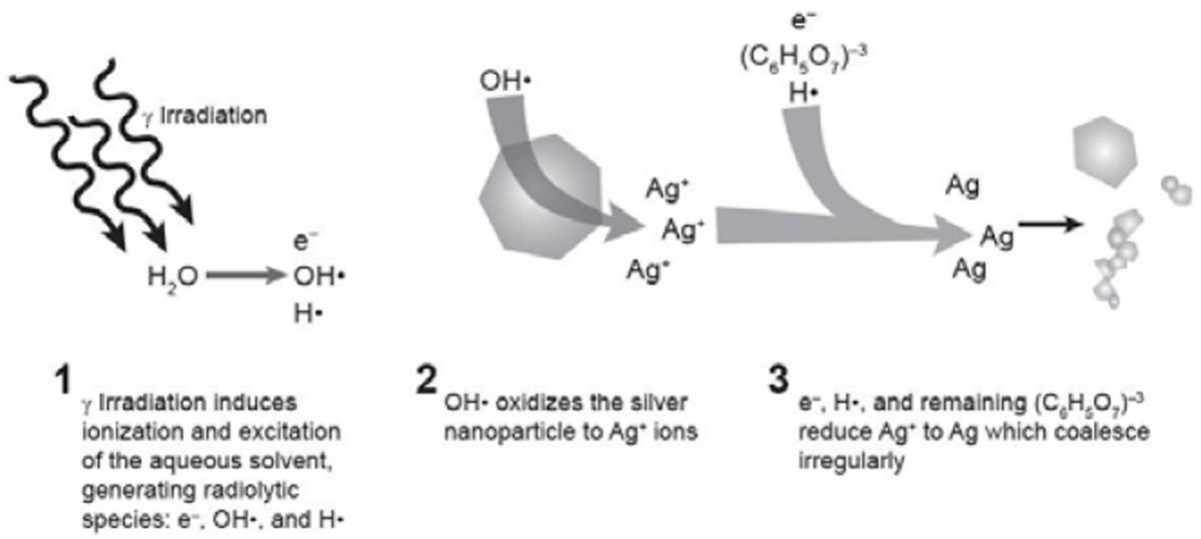
Hypothetical mechanism for morphology change upon exposure to gamma irradiation and EtOx. Hydroxyl radicals produced during gamma irradiation oxidize the silver nanoparticles to Ag+ ions then reducing agents in solution competitively reduce Ag+ ions to Ag atoms. Since there is not sufficient fresh stabilizer in the solution, coalescence is not well controlled, causing the formed particles to display irregular shapes (e.g. the small particles, “ribbon”, “sheet” and “wire” structures we observed).

In light of this, we hypothesized that the hydroxyl radical produced during stabilization by gamma irradiation first oxidizes the silver nanoparticle to Ag^+^ ions. Reducing agents in solution -- including the remaining citrate in the solvent, solvated electrons (e^-^), and hydrogen atoms (H^•^) -- then competitively reduce Ag^+^ ions to Ag atoms, using the residual ablated particles as seed crystals. Since there is not sufficient fresh stabilizer in the solution, coalescence is not well controlled, which would cause all different types of irregular-shaped particle species to be formed -- the small particles and ”ribbon”, “sheet” and “wire” structures observed in (Figure 2). This proposed mechanism is illustrated schematically in Figure 5.

**Figure 6:**
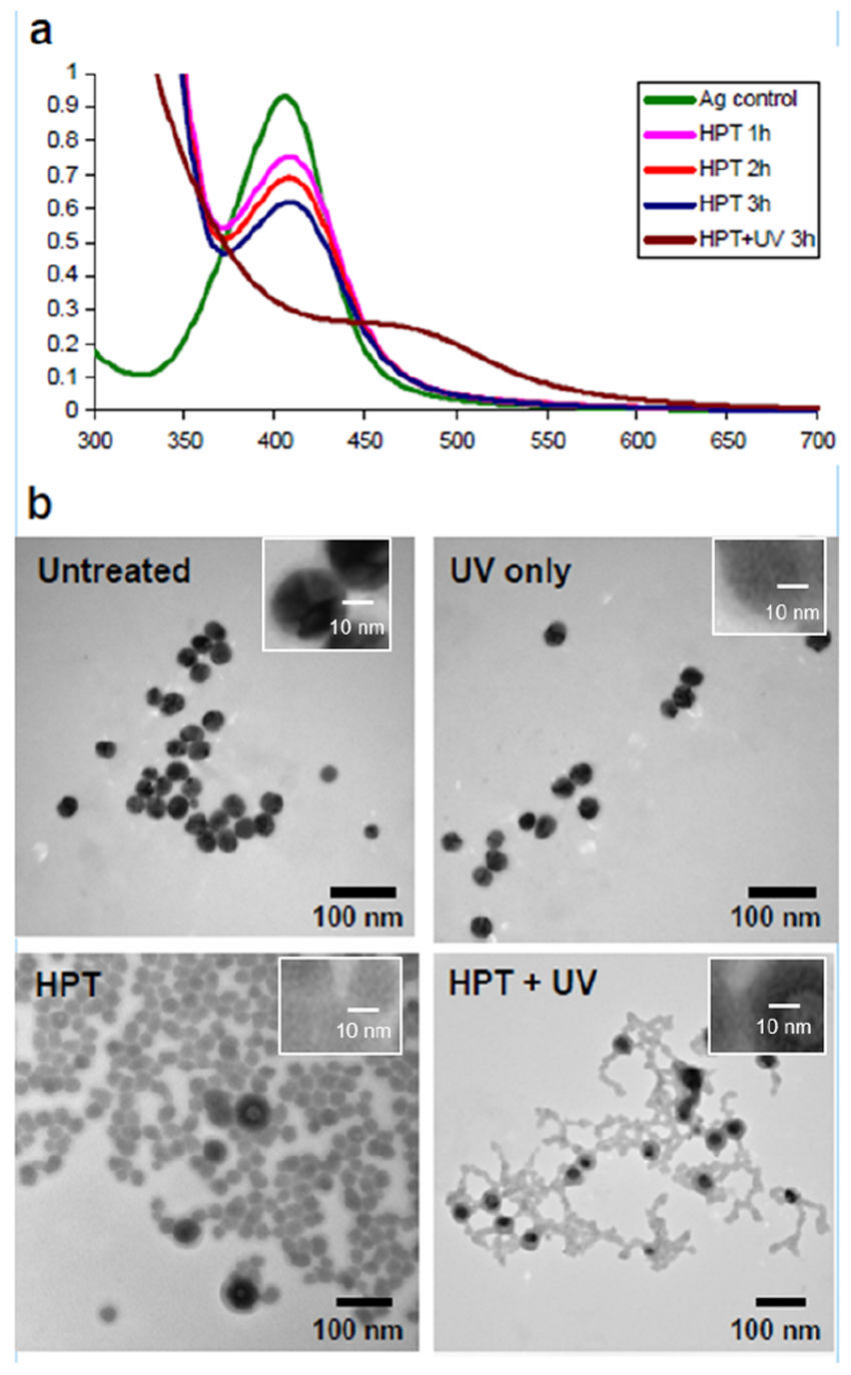
(A) UV-vis spectra of 20 nm silver nanoparticles with treatment of HPT (5 μM), alone or in combination with UV treatment. Addition of UV treatment shows dramatic absorbance differences. (B) TEM images of 20 nm citrate-stabilized silver nanoparticles: untreated (control), treated with UV for 3 hours, treated with HPT (5 μM) for 3 hours, and treated with HPT (5 μM) and UV for 3 hours. From the inserts, it is clear the crystal structure of the silver was destroyed by UV, HPT and HPT + UV treatments.

To test this hypothesis and the free radical mechanism, silver nanoparticles were exposed to N-hydroxy-2-pyridinethione (HPT), which releases hydroxyl radicals under UV or visible light [21]. In addition to TEM, samples in this experiment were also analyzed by UV-Vis spectroscopy, which is more suitable for size-related kinetic studies. As shown in Figure 6a, the plasmon absorbance peak of the silver nanoparticles shows a linear decrease with time upon addition of HPT, even without UV illumination. With the combination of HPT and UV light, the absorbance reveals a pronounced decrease in comparison to the HPT alone, consistent with an increase in hydroxyl radicals and a free radical mechanism. No change was observed in absorbance for the UV treatment of the particles without HPT.

Further evidence of HPT-radical-generated morphology changes are shown in Figure 6b. TEM analysis of the 20 nm silver nanoparticles shows that HPT treatment alone clearly induced degradation of the silver particles, resulting in decreased electron density and the loss of crystal facets on the particle surface. The combination of HPT with UV light generated significantly more morphology changes, forming “wire” structures similar to the long “sheet” structures seen in the gamma irradiated particles.

Thus it appears that at least one of the mechanisms of the instability of silver colloids during irradiation sterilization is the induction of free radical formation by gamma irradiation and scavenging of these radicals by silver. This mechanism may not explain why morphology of silver colloids was changed when samples were dried on TEM smart grids, although it still may be applicable as one cannot exclude the presence of residual moisture in the sample after drying.

## Conclusions

In this study we tested two methods of sterilization traditionally used in medical device industry on silver nanoparticles and monitored the effect on size, morphology and biocompatibility. We observed dramatic changes in size and morphology when the particles were exposed to gamma irradiation, as analyzed by DLS and TEM. We were able to mimic this instability with an inducer of hydroxyl radicals, implying a free radical-based mechanism of action. A four- to five-fold increase in platelet aggregation (*in vitro*) was also observed for gamma irradiated particles, compared to their untreated counterparts. The particles were found to be relatively stable using steam /autoclave sterilization. These data suggest that analysis may be required when selecting a method of sterilization for products containing silver nanoparticles.

## Figures and Tables

**Table 1:** Summary of size analysis of silver nanoparticles before and after sterilization treatments by autoclave and various doses of gamma irradiation.

Sample	Nominal Size (nm)	DLS, Z-Avg (nm)	TEM (nm)

20 nm silver nanoparticles			
untreated	20	34.0 ± 0.4	36.4 ± 5.1

autoclaved	-	33.4 ± 0.2	37.2 ± 5.7

γ- irradiated 15kGy	-	Polydispersed	irregular shape
γ- irradiated 25kGy		Polydispersed	irregular shape
γ- irradiated 50kGy		polydispersed	irregular shape

40 nm silver nanoparticles			
untreated	40	38.3 ± 0.5	55.9 ± 9.6

autoclaved	-	37.8 ± 0.2	54.9 ± 8.4

γ- irradiated 15kGy	-	Polydispersed	irregular shape
γ- irradiated 25kGy		Polydispersed	irregular shape
γ- irradiated 50kGy		polydispersed	irregular shape

60 nm silver nanoparticles			
untreated	60	65.3 ± 0.4	78.3 ± 12.3

autoclaved	-	67.5 ± 1.0	74.6 ± 10.5

γ- irradiated 15kGy	-	Polydispersed	irregular shape
γ- irradiated 25kGy		Polydispersed	irregular shape
γ- irradiated 50kGy		polydispersed	irregular shape

80 nm silver nanoparticles			
untreated	80	90.7 ± 0.3	107.2 ± 14.1

autoclaved	-	90.0 ± 0.6	109.3 ± 13.2

γ- irradiated 15kGy	-	Polydispersed	irregular shape
γ- irradiated 25kGy		Polydispersed	irregular shape
γ- irradiated 50kGy		polydispersed	irregular shape

Note: numbers after the ± symbol represent standard deviations. For the DLS measurements, these values indicate the reproducibility of the measurement (n ≥ 12), not the polydispersity of the size distribution
